# Emerging Computational Methods in Mass Spectrometry Imaging

**DOI:** 10.1002/advs.202203339

**Published:** 2022-10-17

**Authors:** Hang Hu, Julia Laskin

**Affiliations:** ^1^ Department of Chemistry Purdue University 560 Oval Drive West Lafayette IN 47907 USA

**Keywords:** artificial intelligence, computational methods, data‐driven experiments, data mining, mass spectrometry imaging

## Abstract

Mass spectrometry imaging (MSI) is a powerful analytical technique that generates maps of hundreds of molecules in biological samples with high sensitivity and molecular specificity. Advanced MSI platforms with capability of high‐spatial resolution and high‐throughput acquisition generate vast amount of data, which necessitates the development of computational tools for MSI data analysis. In addition, computation‐driven MSI experiments have recently emerged as enabling technologies for further improving the MSI capabilities with little or no hardware modification. This review provides a critical summary of computational methods and resources developed for MSI data analysis and interpretation along with computational approaches for improving throughput and molecular coverage in MSI experiments. This review is focused on the recently developed artificial intelligence methods and provides an outlook for a future paradigm shift in MSI with transformative computational methods.

## Graphical abstract

Computational methods play a key role in mass spectrometry imaging (MSI) by enabling both efficient data analysis and data driven MSI experiments. A concise overview of emerging artificial intelligence methods in MSI provided herein describes challenges associated with the development of these approaches and provides an outlook for a future paradigm shift in MSI enabled by transformative computational methods.

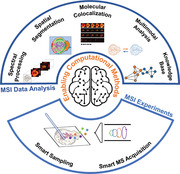

## 1 Introduction

Mass spectrometry imaging (MSI) is a powerful label‐free technique that enables simultaneous imaging of hundreds of molecules in biological samples with high sensitivity and unprecedented molecular specificity.^[^ ^1^, ^2^, ^3^, ^4^, ^5^, ^6^, ^7^ ^]^ Many classes of biomolecules including metabolites, lipids, peptides, proteins, and drugs have been characterized using MSI. In the past four decades, MSI technologies underwent impressive developments through continuous advances in both soft ionization techniques and mass spectrometry (MS) instrumentation. Dramatic improvements in the spatial resolution, molecular coverage, quantification capabilities, and throughput have established MSI as a powerful tool in biological research, clinical studies, drug discovery, and other fields.^[^ ^8^, ^9^, ^10^ ^]^

MSI experiments are performed by acquiring mass spectra over a virtual grid of pixels on a sample surface. In these experiments, a complex mixture of molecules is removed from the sample and ionized for MS analysis.^[^ ^1^, ^2^, ^3^, ^7^, ^8^ ^]^ Numerous desorption/ionization techniques in which analyte molecules are desorbed using a laser beam, cluster beam, charged microdroplets, or small liquid volume have been coupled with MSI. Typical MSI experiment generates hundreds of thousands of mass spectra, each containing thousands of distinct *m*/*z* features. These features are subsequently extracted and visualized as 2D heat maps, which depict spatial distributions of molecules in the sample.

Advances in improving the spatial resolution^[^ ^11^, ^12^, ^13^, ^14^ ^]^ and acquisition throughput^[^ ^15^, ^16^, ^17^, ^18^ ^]^ of MSI result in a substantial increase in data size, which necessitates the development of more efficient computational tools for data visualization and analysis. Furthermore, multimodal imaging, which combines one or more imaging modalities with MSI, is also gaining increasing popularity. Additional modalities usually provide complementary topographical or molecular information thereby enhancing the information content of imaging experiments.^[^ ^19^, ^20^ ^]^ The interpretation of both MSI and complex multimodal data, which relies on advanced computational methods for data mining and visualization, is a major bottleneck for deriving biological conclusions from the rich imaging data.^[^ ^21^, ^22^, ^23^ ^]^ In addition, computationally driven MSI experiments present a new paradigm for further improving the MSI throughput and molecular coverage with little or no hardware modifications.

**Figure 1:**
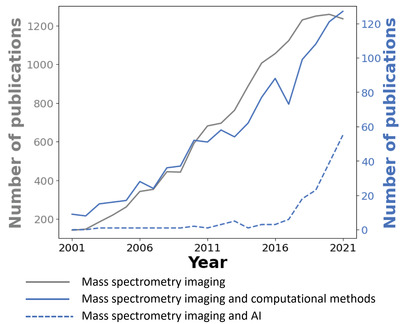
Number of publications focused on MSI and computational methods in MSI obtained by searching Web of Science.

In this review, we provide a concise overview of new computational methods in MSI and discuss challenges associated with the development of these approaches. For the experimental aspects of MSI, readers are referred to a collection of review articles^[^ ^1^, ^2^, ^3^, ^4^, ^5^, ^6^, ^7^, ^8^, ^9^, ^10^ ^]^ with detailed summaries and illustrations of the ionization methods, sample preparation, and experimental workflows. **Figure** **1** provides a summary of the number of publications on the Web of Science database related to MSI and computational tools in this field. Gray curve shows the total number of publications obtained by searching for “mass spectrometry” and “imaging”. Blue curve shows the number of publications obtained by adding the following keywords to the search: “statistic*”, “computation*”, “machine learning”, “deep learning”, and “artificial intelligence”. Meanwhile, blue dashed curve highlights a subset of publication in this category that includes only “machine learning”, “deep learning”, and “artificial intelligence”. The results shown in Figure 1 highlight the exponential growth in the development of modern computational tools based on artificial intelligence (AI) in the past five years (blue dashed line).

This review aims to provide MSI experimentalists with a roadmap for selecting state‐of‐the‐art computational tools for their research. First, we describe several computational workflows for MSI data analysis, including spectral data processing, data compression, statistical analysis, multimodal analysis, and data interpretation using a reference knowledge base. We focus on emerging AI approaches, which have already shown superiority for handling vast MSI data. Next, we provide an overview of the open‐source software and community resources in this field. Finally, we highlight computation‐guided data acquisition approaches as an enabling tool for improving MSI experiments. We conclude by providing a critical outlook for future developments of transformative computational tools.

## 2 Computational Methods for MSI Data Analysis

**Figure 2:**
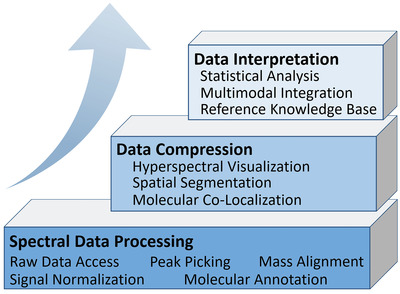
Information flow in three levels of MSI data analysis.

**Figure** **2** summarizes three levels of data analysis necessary for deriving biological insights from complex MSI data. The first step involves spectral data processing (bottom level), in which molecular features at a pixel level are extracted from the experimental data for downstream analysis. After preprocessing, MSI data can be organized as a 3D data cube,^[^ ^24^ ^]^ which has high dimensionality in both the spectral and spatial domains. The second level shown in the middle summarizes data compression by dimensionality reduction and classification of either spectral or spatial information. This analysis enables spatial segmentation or molecular colocalization, which links the spectral and spatial information to provide a concise representation of the vast MSI data. The final level of data analysis employs statistical tools to infer biological insights from MSI data.^[^ ^25^, ^26^ ^]^ The statistical analyses are often coupled with information provided by other imaging modalities, which places the spatial molecular information into the context of anatomical regions and/or provides a complementary chemical characterization of the sample.^[^ ^23^ ^]^ Furthermore, molecular colocalizations may be rationalized using reference knowledge base‐enabled ontology and pathway analysis.

### 2.1 Spectral Data Processing

Spectral data processing is a fundamental step for accurately reporting the identity and quantity of molecules observed in MSI data. A typical workflow involves raw data access, baseline correction, peak picking and selection, mass alignment, signal normalization, and molecular annotation. Many well‐established computational tools in the MS community have been used for MSI data processing. Several review articles provide an in‐depth summary of the spectral data analysis strategies.^[^ ^21^, ^22^ ^]^ Herein, we focus on the recent developments in data processing.

#### 2.1.1 Raw Data Access

MSI experiments are performed using a wide range of mass spectrometers with different vendor‐specific data formats. Accessing binary MS data files is an important step in the MSI data analysis pipeline. Fortunately, with the emerging trend of open science, most instrument vendors provide binary libraries for users to extract information from raw data files. In addition, many open‐source tools for raw MS data access have been developed for programming ecosystems including Python, R, and C#. An important breakthrough was achieved a decade ago when ProteoWizard integrated vendor data reader libraries independently of instrument controls and allowed direct MS data extraction by C# and R.^[^ ^27^ ^]^ In 2017, a Python‐based ecosystem multiplierz v2.0 was developed.^[^ ^28^ ^]^ It provided a Python application programming interface (API) for accessing raw data in common vendor data formats, including .d for Agilent and Bruker Daltonics, .wiff/.t2d for SCIEX, and .RAW for Thermo Fisher. More recently, OpenTIMS^[^ ^29^ ^]^ and AlphaTims^[^ ^30^ ^]^ have been developed to access data from Bruker Daltonics trapped ion mobility spectrometer (TIMS) and hybrid quadrupole‐time‐of‐flight mass analyzer using Python and R. In addition, a vendor neutral MSI data format, imzML,^[^ ^31^ ^]^ has been developed for MSI data exchange and adopted in major repositories (e.g., METASPACE^[^ ^32^ ^]^) and biomolecular atlas programs, such as HuBMAP.^[^ ^33^ ^]^

#### 2.1.2 Peak Picking

Peak picking involves either peak detection or binning of the *m/z* range to convert a mass spectrum into a list of peaks with centroid *m*/*z* values and associated intensities. Peak picking effectively distinguishes molecular features from MSI background and noise signals, which significantly reduces data size. A combination of baseline correction, spectral smoothing, and peak picking is routinely performed for MALDI‐TOF MSI data as discussed in a review paper by Rafols et al.^[^ ^21^ ^]^ A majority of peak picking methods involve detection of local maxima in the mass spectrum or fitting peaks with Gaussian functions.^[^ ^34^ ^]^ More recently, wavelet transform based approaches have been developed for accurately identifying and separating MS signals from noise and baseline.^[^ ^35^, ^36^ ^]^ In addition, Lieb et al.^[^ ^37^ ^]^ have developed a method using sparse approximations of frame multipliers and including MSI spatial information, which helps detect spatially adjacent high‐ and low‐abundance peaks and eliminates baseline contribution and noise signals. After peak picking, peaks of biological interest are selected for downstream analysis.^[^ ^38^ ^]^

#### 2.1.3 Mass Alignment

**Figure 3:**
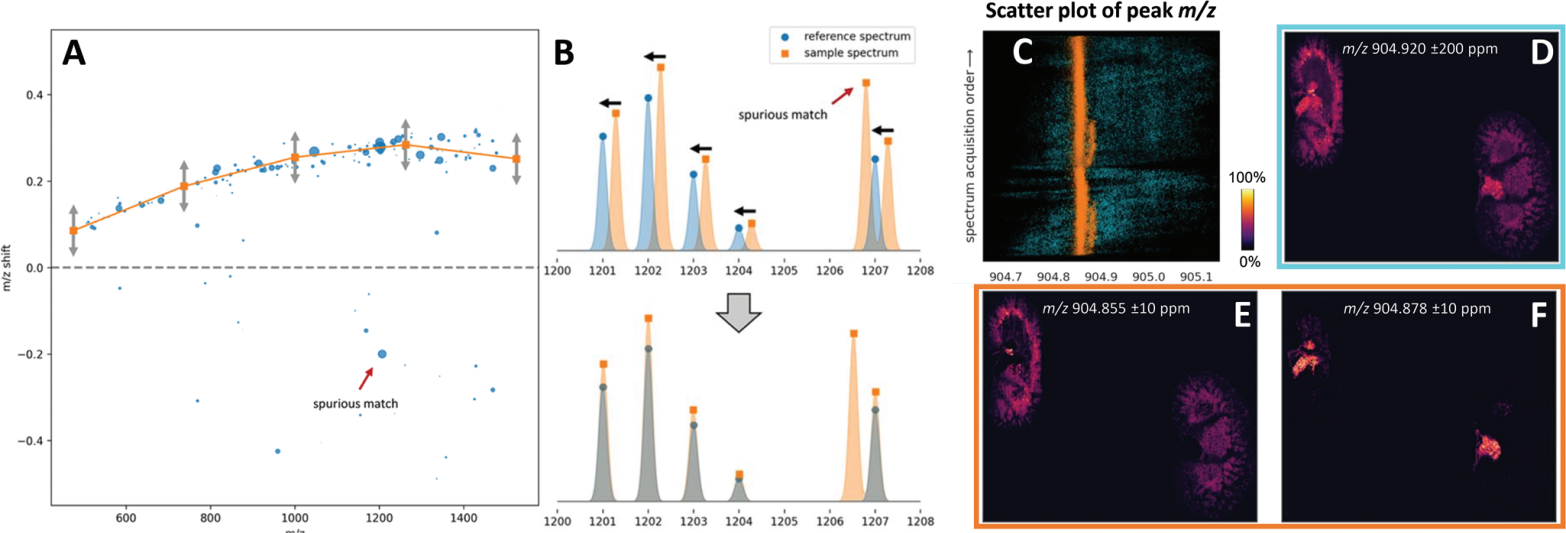
Spectral alignment using COW algorithm. A) The matching of peaks in the sample spectrum against the reference spectrum. Blue scatters show the mass shift of peaks in a sample spectrum and orange curve shows local mass warpings. B) Zoom‐in of the matching between sample and reference spectra with centroid peaks modeled as Gaussians. C) Scatter plot of peak *m*/*z* values before (cyan) and after (orange) spectral alignment at a small *m*/*z* region for a TOF kidney data. Ion images generated from D) original spectra and E,F) aligned spectra. Reproduced with permission.^[^ ^44^ ^]^ Copyright 2020, American Chemical Society.

Mass alignment between pixels is a critical first step toward generating ion images. Shifts in the *m*/*z* values between pixels are common in MSI data and become more severe when lower resolution mass analyzers are employed. To address this issue, signals of known ions, such as internal standards or matrix‐related ions in MALDI, have been used to recalibrate mass spectra.^[^ ^39^, ^40^, ^41^ ^]^ Many computational methods have been developed for spectral alignment in the postacquisition data processing step to reduce mass shifts.^[^ ^42^, ^43^, ^44^ ^]^ Eriksson et al.^[^ ^44^ ^]^ used a correlation optimized warping (COW) algorithm to reduce mass shifts in MSI data by warping the mass dimension without standards. In this approach, each mass spectrum is recalibrated by maximizing its similarity to the reference spectrum as shown in **Figure** **3**A. The similarity of two centroid spectra is obtained by modeling each peak as a Gaussian function and calculating the sum of all pairwise peak overlaps (Figure 3B). In addition to *m*/*z* location, height, and peak width, the spatial information has been also utilized to further improve the quality of mass alignment. This approach effectively reduces *m*/*z* shifts across the MSI data postacquisition. In Figure 3C, peak *m*/*z* values extracted from the entire MSI data set before and after spectral alignment are shown in cyan and orange colors, respectively. COW calibration enables the identification and separation of two adjacent peaks eliminating the interference shown in Figure 3D. Using a narrow mass window, recalibrated MSI data generate two ion images of these features with distinct spatial distributions (Figure 3E,F). In another approach, signals of known background ions and endogenous lipids observed in MSI experiments have been used as calibrants.^[^ ^45^, ^46^ ^]^ For example, Rocca et al.^[^ ^46^ ^]^ developed a method by mining *m*/*z* values of endogenous calibrants from publicly available MSI data repository. By searching a large number of datasets, they generated an *m*/*z* list for candidate endogenous biological signals. Then, they calculated and selected matches between the experimental peaks and candidate calibration signals. A random sample consensus linear model was used on the match searches to calibrate spectral *m*/*z* values.

#### 2.1.4 Signal Normalization

Signal normalization is used to remove artifacts from molecular images, which originate either from matrix effects or experimental variations in MSI sampling and ionization.^[^ ^10^ ^]^ Several normalization methods have been used to improve the visualization and quantification of MSI data. Signal normalization to the total ion current (TIC) is the most widely used approach. Normalization to the signal of an internal standard is typically used in quantitative MSI experiments.^[^ ^47^, ^48^, ^49^ ^]^ Computational methods have also been developed to improve signal normalization. Veselkov et al.^[^ ^50^ ^]^ introduced a median fold change (MFC) normalization to remove biologically unrelated pixel‐to‐pixel variation using median peak intensity in each spectrum. More recently, Boskamp et al.^[^ ^51^ ^]^ developed an intensity profile normalization (IPN) approach, which improves site‐to‐site reproducibility for interlab and cross‐protocol comparisons. This approach computes spectral intensity profiles as a function of *m*/*z* values for each pixel and normalizes them using a reference profile, which enables nonlinear and mass‐dependent intensity transformation for each peak. They also benchmarked a range of normalization approaches, including TIC, MFC, and IPN for tumor classification. In this test, IPN provided the best classification performance. Song et al.^[^ ^52^ ^]^ reported a distinct strategy for the normalization of MS signals for selected molecules of interest. Based on the supervised machine learning, they established a quantitative relationship between the absolute concentration of a drug and endogenous metabolite signals in various tissue microenvironments. They used homogenized tissue samples with spiked drug molecules as ground‐truth data to construct regression models. These models were used for computational normalizations, which directly converted peak intensities of endogenous metabolites in each pixel into drug concentrations. In addition, Pace et al.^[^ ^53^ ^]^ applied a sequential paired covariance (SPC) approach to transform ion intensities for improving ion image visualization. Compared to TIC and MFC, the SPC approach reduces the inter‐pixel variability and preserves distinguishable biological features.

#### 2.1.5 Molecular Annotation

Molecular annotation is critical to deriving biological conclusions from MSI data. In conventional MSI experiments, peak annotation is carried out based on accurate mass of intact ions and tandem mass spectrometry (MS/MS) data acquired either directly on a tissue or using off‐line bulk analysis. More recently, ion mobility spectrometry has been coupled with MSI to facilitate isomeric and isobaric separation and obtain additional structural information for analyte identification.^[^ ^8^, ^9^ ^]^ Moreover, MS/MS imaging has been used for the simultaneous imaging and identification of biomolecules in tissues.^[^ ^54^, ^55^ ^]^ Several powerful community resources have been developed to enable identification of metabolites,^[^ ^32^, ^56^, ^57^, ^58^, ^59^ ^]^ lipids,^[^ ^60^, ^61^, ^62^, ^63^ ^]^ glycans,^[^ ^64^, ^65^ ^]^ and proteins^[^ ^66^, ^67^, ^68^, ^69^ ^]^ based on accurate mass, MS/MS, or collision cross‐section (CCS) measurement.

Database searching and scoring algorithms have been developed to support this strategy. For example, Li et al.^[^ ^70^ ^]^ benchmarked 42 MS/MS similarity metrics for metabolites present in the NIST20 library and demonstrated that the spectral entropy similarity score provided the best matching performance in terms of the false discovery rate (FDR). Palmer et al.^[^ ^71^ ^]^ developed an FDR‐controlled metabolite annotation approach specially for MSI data. This method uses a joint match score, which involves accurate mass, isotopic pattern, and spatial distribution to improve the search performance. Meanwhile, rMSIannotation^[^ ^72^ ^]^ employs isotopic pattern coherence, image correlation, and mass error to annotate isotopic and adduct ions in MSI data. Other than small molecule annotations, on tissue top‐down proteomics has been used for the annotation of intact proteins observed in nanospray desorption electrospray ionization (nano‐DESI) MSI experiments based on MS/MS data.^[^ ^73^, ^74^ ^]^ For example, Yang et al.^[^ ^74^ ^]^ used ProSight software and a Rattus Norvegicus database to annotate proteoforms observed using nano‐DESI MSI of mouse brain tissues.

Molecular annotation approaches that do not involve database searching classify analytes observed in MSI experiments based on the accurate mass, MS/MS, or CCS measurement. For example, Kendrick mass defect (KMD) analysis has been used for grouping lipids and metabolites into molecular classes by identifying homologous series based on mass defect.^[^ ^75^, ^76^ ^]^ Using a 2D KMD plot, peaks in MSI data have been grouped into classes of lipopeptides, surfactins, lipids, and polymers.^[^ ^75^ ^]^ Different classes of lipids can be further distinguished using a reference KMD approach.^[^ ^76^ ^]^ In addition, ion images with MS/MS data may be organized into molecular networks based on the similarity in fragmentation patterns, which has been used for identifying unknowns in biological samples by comparing their fragmentation with MS/MS of previously reported compounds.^[^ ^77^ ^]^ Meanwhile, CCS measurements have been used to classify lipids based on the observed linear trend in CCS versus *m*/*z* for the same lipid class and type of adduct formed in ionization.^[^ ^78^ ^]^

### 2.2 Data Compression

**Figure 4:**
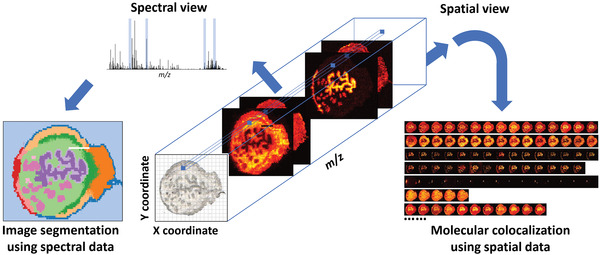
Data compression of an MSI data cube obtained for a mouse uterine tissue section using image segmentation and molecular colocalization. Image segmentation classifies pixels based on spectral similarity and molecular colocalization classifies molecules based on the distribution (image) similarity. Although only four ion images are shown in the data cube to illustrate the correlation between the spectral and spatial data, the results of data compression are based on hundreds of ion images. Adapted with permission.^[^ ^83^ ^]^ Copyright 2021, American Chemical Society.

After spectral processing, MSI data may be represented as a 3D data cube with high dimensionality as shown in **Figure** **4**.^[^ ^24^ ^]^ In this example, a nano‐DESI MSI dataset of a mouse uterine tissue section, which contains several distinct cell types distributed over a small cross‐section, is selected as a model system.^[^ ^14^ ^]^ Data compression is used to concisely represent MSI data while retaining important spectral and spatial information. It is also an essential step in the MSI machine learning pipeline. Methods for MSI data compression can be grouped into two strategies: dimensionality reduction and classification. Several key analysis results from data compression include image segmentation and molecular colocalization as shown in Figure 4. They provide a direct connection between the spectral and spatial information, which is critical for deriving biological insights from MSI data. Herein, we summarize recent advances in computational methods for data compression of complex spectral and spatial data.

#### 2.2.1 Data Compression of Spectral Data Using Unsupervised Methods

In MSI, a complete mass spectrum containing signals of thousands of molecules is acquired in each pixel. The complexity of MSI data leads to the so‐called “curse of dimensionality” for computational algorithms. Indeed, in the high‐dimensional space, data becomes extremely sparse and the underlying features tend to be masked by noise signals. Dimensionality reduction techniques address this issue by selecting a subset of features or transforming the original data into a lower‐dimensional space while retaining meaningful information.^[^ ^79^ ^]^ Transformation‐based unsupervised dimensionality reduction approaches, which focus on discovering the underlying structure in MSI data without requiring prior knowledge, are widely applied in exploratory analysis. The readers are referred to a comprehensive review of unsupervised machine learning algorithms and their applications in MSI data analysis, which will not be discussed herein.^[^ ^80^ ^]^ Instead, we briefly introduce the most popular methods and focus on the most recent approaches in this field.

**Figure 5:**
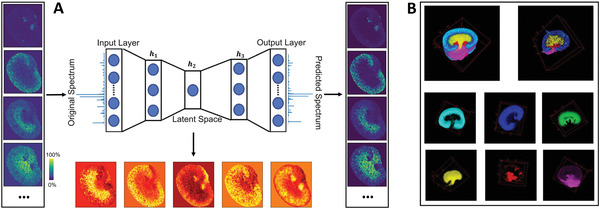
A) Neural network architecture of msiPL. An autoencoder configuration enables the model to predict spectra and learn spectral representations. Representative experimental ion images (left column) and predicted ion images (right column) demonstrate that MSI peaks were learned by this model. In addition, 5D encoded features (bottom row) capture molecular structures in the original high‐dimensional data. B) Image segmentation maps of a 3D MALDI MSI dataset obtained by coupling msiPL feature encoding and GMM clustering. Adapted under the terms of the Creative Commons Attribution 4.0 International License.^[^ ^98^ ^]^ Copyright 2021, The Authors, published by Springer Nature.

From the early days, matrix factorization methods, such as principal component analysis (PCA),^[^ ^81^, ^82^, ^83^ ^]^ non‐negative matrix factorization (NMF),^[^ ^84^, ^85^ ^]^ and multivariate curve resolution‐alternating least squares (MCR‐ALS)^[^ ^86^, ^87^ ^]^ have been widely used in MSI. Nonlinear manifold learning methods, such as self‐organized maps (SOM),^[^ ^88^, ^89^, ^90^ ^]^ t‐distributed stochastic neighbor embedding (t‐SNE),^[^ ^91^, ^92^, ^93^ ^]^ and uniform manifold approximation and project (UMAP)^[^ ^94^, ^95^ ^]^ have gained popularity due to their ability to preserve the local structure of the high‐dimensional data in the low‐dimensional visualization. In recent years, self‐supervised learning has gained momentum as an efficient approach that eliminates laborious data labeling for training neural networks. In this strategy, the original data provides a supervisory signal that enables training of the model without manual annotation. An autoencoder is one of the self‐supervised learning algorithms, which trains a neural network encoder for dimensionality reduction and a decoder for generating synthetic signals.^[^ ^96^ ^]^ Thomas et al.^[^ ^97^ ^]^ pioneered the use of an autoencoder for reducing the dimensionality of spectral data. Abdelmoula et al.^[^ ^98^ ^]^ extended this strategy by developing an msiPL framework to encode MSI data and bypass spectral preprocessing as shown in **Figure** **5**A. They demonstrated that msiPL effectively learned a 5D representation at a hidden layer h_2_, which concisely depicts molecular distributions in the original high‐dimensional data. Unlike t‐SNE and UMP which load full‐size data into the RAM, the neural network framework uses an iterative computation with data split into minibatches, which allows msiPL to handle extremely large 3D MSI data using reasonable RAM resources.

Unsupervised dimensionality reduction is often used for hyperspectral visualization, which displays the spectral correlation between pixels using multiple colors in a single image. Manifold learning approaches, such as t‐SNE,^[^ ^99^ ^]^ SOM,^[^ ^89^, ^90^ ^]^ and UMAP^[^ ^94^ ^]^ provide powerful visualization capabilities in an extremely low‐dimensional space. They compress mass spectra and encode MSI information into 3D data, which are subsequently visualized using either RGB or CIELab color schemes. This visualization reveals distinguishable anatomical regions of tissue samples using color gradients, which enables histology‐like analysis. Sarycheva et al.^[^ ^100^ ^]^ further combined the advantage of matrix factorization (NMF) and manifold learning (t‐SNE) to generate hyperspectral visualizations. In a CIELab color scheme, they applied a structure visualization method to encode multiple NMF component images into an L channel for lightness and used t‐SNE 2D embedding as a and b channels for chromaticity. Experiments on both simulated and experimental data show that this new strategy improves the visual perception of tissue heterogeneity.

Following unsupervised dimensionality reduction, multivariate clustering is performed to classify pixels and spatially partition the image. The resulting spatial segmentation map provides biologically relevant regions of interest (ROIs) in either tissue or cell samples. ROIs are used both for data visualization and quantitative analysis of tissue microenvironments. Hierarchical clustering (HC),^[^ ^101^ ^]^ k‐means,^[^ ^81^, ^82^, ^87^ ^]^ Gaussian mixture model (GMM),^[^ ^83^, ^98^ ^]^ spectral clustering,^[^ ^102^ ^]^ and density‐based clustering^[^ ^95^ ^]^ have been applied to MSI data. The principles of these algorithms are described in detail in the review paper.^[^ ^80^ ^]^ Briefly, clustering algorithms classify pixels based on spectral similarity. The dimensionality reduction step is usually performed before clustering because the performance of similarity measurements deteriorates in the high‐dimensional space. Features generated by matrix factorization techniques, such as PCA, NMF, and MACR‐ALS, preserve the relative distances of the high‐dimensional spectral data and work well in combination with k‐means clustering,^[^ ^81^, ^82^, ^87^ ^]^ which detect cluster communities based on distance measurements. Meanwhile, manifold learning techniques preserve local data structure and are more suitable for density‐based clustering methods, such as HDBSCAN.^[^ ^95^ ^]^ In addition, distribution‐based clustering approaches, such as GMM, have demonstrated excellent robustness for clustering of MSI data after both linear and nonlinear dimensionality reduction.^[^ ^83^, ^98^ ^]^ For instance, GMM has been coupled with msiPL to perform segmentation of a 3D MALDI MSI data. As shown in Figure 5B, 5D features from MSI datasets of 72 sequential mouse kidney tissue sections were obtained using msiPL and clustered into anatomical 3D structures using GMM. Several novel methods and algorithms have been developed to incorporate spatial information into spectral similarity to improve the segmentation results. One strategy is to include an ion image denoising process to reduce the interpixel spectral variation before clustering.^[^ ^102^, ^103^ ^]^ Another strategy is to exploit spatially‐aware distance measurement in clustering algorithms, which takes into account spatial neighborhoods when computing spectral similarity.^[^ ^104^, ^105^ ^]^ Despite many successful applications, spatial segmentation results generated by multivariate clustering do not necessarily capture all the patterns in MSI data because of the inevitable information loss in the dimensionality reduction step. A univariate analysis using clustering or thresholding may be used to generate complementary ROIs from single ion images.^[^ ^83^, ^106^ ^]^ After obtaining ROIs from the spatial segmentation, morphological operations, such as opening, closing, and erosion, may be further applied to clean or select spatial features from binary ROI images.^[^ ^107^, ^108^ ^]^

In summary, unsupervised data compression of MSI spectral data using dimensionality reduction and clustering analysis provides concise representations of the molecular heterogeneity in biological samples. The combination of compressed spectral data and spatial information enables the identification of different cell types and their localization in the sample. These approaches identify ROIs for further quantitative and statistical analyses.

#### 2.2.2 Compression of Spectral Data Using Supervised Methods

**Figure 6:**
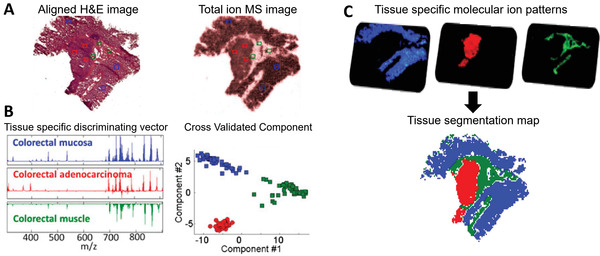
Supervised MS multivariate analysis and classification for colorectal cancer tissues. A) Spatially aligned H&E image and total ion MS image. The locations where mass spectra were selected for RMMC modeling are marked with colors. B) RMMC multivariate analysis reveals tissue specific discriminating vectors and generates components for efficient transformation of MSI data. C) In the spatial domain, RMMC analysis provides tissue specific molecular ion patterns. Tissue class annotation is also obtained by k‐nearest‐neighbor classification of RMMC features. Adapted with permission.^[^ ^125^ ^]^ Copyright 2014, National Academy of Sciences.

Supervised data compression methods are used when predefined classifications of different regions in the sample are available. In these methods, spectral dimensionality reduction is usually performed together with pixel classification. This strategy focuses on either enhancing the classification capability for new data or interpreting the classification models. For example, least absolute shrinkage and selection operation (Lasso) has been used to classify tumor regions based on MSI data.^[^ ^109^, ^110^, ^111^, ^112^, ^113^ ^]^ Lasso is a multiclass‐logistic regression method, which performs both feature selection and L1 regulation in order to enhance the prediction accuracy and model interpretability based on molecular information. Using Lasso, Margulis et al.^[^ ^110^ ^]^ identified 24 characteristic ion signals of lipids and metabolites in DESI MSI data of human surgical specimens, which yielded a 94.1% diagnostic accuracy for tumor aggregates of basal cell carcinoma. Among the identified species, fumarate, which is a Krebs cycle intermediate, has been verified as a biomarker of aberrant tumor metabolism. In addition, recursive feature elimination^[^ ^114^ ^]^ and gradient boosting tree ensemble^[^ ^115^ ^]^ algorithms have been used to select ion features from mass spectra and classify MSI pixels. Partial least‐squares (PLS) regression,^[^ ^116^, ^117^, ^118^ ^]^ linear discriminant analysis (LDA),^[^ ^119^, ^120^, ^121^, ^122^ ^]^ and recursive maximum margin criterion (RMMC)^[^ ^123^, ^124^, ^125^, ^126^, ^127^ ^]^ are most popular algorithms for linear transformation. The objective of PLS is to maximize th between‐class variance. By contrast, LDA components are derived by maximizing the ratio of between‐versus within‐class variance. However, LDA cannot be applied to data in which the number of variables exceeds the number of samples. To bypass this limitation, the PCA–LDA analysis is performed by incorporating PCA as a preprocessing step. Similar to PCA–LDA, RMMC simultaneously maximizes the difference between interclass and intraclass variability and eliminates manual selection of an optimal number of PCA components.^[^ ^123^ ^]^ When applied to MSI data, these algorithms use pixel classifications as a reference to linearly project spectral data into a lower‐dimensional space for distinguishing tissue subregions. By interpreting the projection coefficients of features in transformed components, one can identify key molecules characteristic of specific regions in a sample. For example, Mei et al.^[^ ^118^ ^]^ used the PLS algorithm to correlate secondary ion mass spectrometry (SIMS) imaging data to the colony‐formation frequency in the evaluation of biomaterials for human stem cell growth. Based on the variable coefficients in the PLS model, they identified important functional components of biomaterials which promoted colony formation. Veselkov et al.^[^ ^125^ ^]^ used the RMMC approach to extract tissue‐type‐specific MS signatures for colorectal cancer as shown in **Figure** **6**. In this chemoinformatic strategy, the optical H&E staining image is aligned with the total ion MS image (Figure 6A), which enables selecting mass spectra from cancer, muscle, and mucosa tissue regions for training of the RMMC model. The RMMC‐derived components reveal tissue‐type‐specific chemical signatures and differentiate tissue classes in the feature space (Figure 6B), which are then displayed in the spatial context of the tissue as shown in Figure 6C.

Nonlinear supervised learning models such as support vector machine (SVM),^[^ ^128^, ^129^, ^130^ ^]^ random forest (RF),^[^ ^131^, ^132^, ^133^ ^]^ and deep learning^[^ ^134^, ^135^, ^136^, ^137^, ^138^, ^139^ ^]^ are also widely used for classifying spectral data for spatial segmentation. The SVM algorithm finds a hyperplane with maximum margin to classify data. A kernel function is used to nonlinearly map the learning data into a higher dimensional feature space, which makes them more separable.^[^ ^140^ ^]^ By contrast, RF is an ensemble classifier that trains a number of decision tree models on various subsets of the data, which reduces the risk for overfitting of high‐dimensional data.^[^ ^141^ ^]^ Both SVM and RF are well established methods commonly implemented in MSI data analysis software. Their low computational cost makes them compatible with personal computers. Recently, deep learning methods have gained increasing popularity because of their superior classification accuracy and ability to process large amounts of MSI data. Many deep learning architectures have been developed based on the fully connected neural networks,^[^ ^139^ ^]^ convolutional neural network (CNN),^[^ ^134^, ^136^, ^137^, ^138^ ^]^ and recurrent neural network (RNNs),^[^ ^135^ ^]^ which provide better spectral classification accuracy than conventional machine learning methods. The 1D convolution computation in CNN architectures has been demonstrated as a promising approach to extract and digest molecular information from correlated mass spectral information. For example, Behrmann et al.^[^ ^134^ ^]^ used convolution kernels of size 3 with small receptive filed to learn isotopic patterns in IsotopeNet. Van Kersbergen et al.^[^ ^136^ ^]^ employed dilated convolutions to capture both locally and globally distributed spectral patterns. Although deep learning approaches have demonstrated their potential for supervised image segmentation, the limited size of training MSI data is currently a major bottleneck to their utilization. Several strategies have been developed to address this challenge. First, multiple manually annotated datasets have been used to train the same representation learning model in a supervised manner. Seddiki et al.^[^ ^138^ ^]^ have developed a cumulative learning strategy to successively train the same CNN model with MSI datasets acquired from different organisms. As a result, this workflow optimized the CNN model parameters for learning spectral representations from distinct MSI data. In another approach, either an unsupervised or self‐supervised learning is adopted as a preprocessing step to train a representation learning model with unlabeled data. Based on this model, a classification layer is appended, and a final classification model is further trained using a small amount of manually labeled data. Following the success of msiPL, Abdelmoula et al.^[^ ^139^ ^]^ developed massNet, which uses the msiPL autoencoder to train the neural network for learning the representations of mass spectra. Using the pretrained encoder, they trained a classification model to distinguish tumor from healthy tissues.

#### 2.2.3 Data Compression for Spatial Data

Although compression of spectral data is a powerful approach for revealing the molecular heterogeneity of tissue samples in the spatial domain, it does not provide an overview of the spatial patterns observed in MSI experiments. In addition, molecular colocalization, in which one ion signal is spatially associated with other ions or reference patterns, is critical to the identification of biochemical pathways for biomarker discovery, drug development, and clinical diagnostics. Sorting and classifying ions in MSI data with respect to their spatial distributions is an important task that has attracted considerable attention. Spatial data compression provides solutions for this type of analysis. However, the development of computational methods has not made a significant progress until recent breakthroughs in manifold learning and deep learning. In particular, deep learning enables the identification of high‐level features in the spatial domain, which substantially improves its ability to generate image representations in comparison with traditional data compression approaches. To draw an analogy to the field of computer vision, although many machine learning algorithms had been tested, including PCA, LDA, SVM, and RF, the recurring image recognition challenge has been addressed only recently using deep CNN.^[^ ^142^ ^]^

In the early spatial data compression methods, 2D ion images are converted into 1D vectors and several similarity measurements of vectors including Euclidean distance, Pearson correlation coefficient, cosine similarity, and hypergeometric similarity are used to determine molecular colocalizations.^[^ ^143^, ^144^, ^145^, ^146^, ^147^, ^148^ ^]^ Based on the pairwise similarity measures, an adjacency matrix is computed for constructing a molecular network, which enables the detection and visualization of colocalized ions.^[^ ^145^, ^146^ ^]^ In addition, unsupervised classification approaches for ion images have been developed using dimensionality reduction (PCA and UMAP) and clustering (k‐mean, HC, GMM, and HDBSCAN).^[^ ^83^, ^95^, ^149^, ^150^, ^151^, ^152^, ^153^ ^]^ However, these methods cannot effectively correlate high‐level spatial features based on 1D vectors making them disproportionately sensitive to the experimental artifacts and noise. Image similarity metrics established by the computer vision community including structure similarity index, multiscale multifeatured similarity index, and morphometric analysis have also been used to quantify molecular colocalizations in MSI data.^[^ ^148^, ^154^, ^155^ ^]^ More recently, CNN models have gained popularity for exploring molecular colocalizations. For example, Zhang et al.^[^ ^156^ ^]^ have developed a transfer learning approach to extract spatial features from ion images using a pretrained Xception CNN model. In the clustering analysis, this approach outperformed UMAP and provided a better classification of ion images. Ovchinnikova et al. approached this challenge using supervised learning. They created a large amount of gold standard ion images that are manually organized by experts from METASPACE knowledge base. Based on these ground‐truth data, they developed a deep learning‐based Pi model to quantify colocalization between ion images.^[^ ^147^ ^]^ They also used a labeled dataset to retrain an ResNet‐50 CNN model to identify off‐sample ion images with a recall score of 98%.^[^ ^157^ ^]^

**Figure 7:**
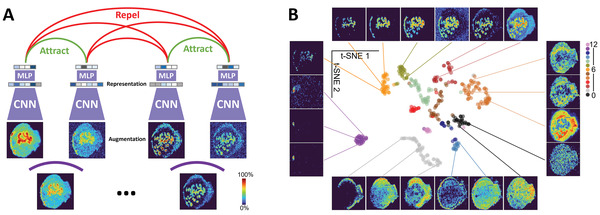
A) SimCLR framework for training of a CNN model for learning ion image representations without annotations. B) A t‐SNE plot of learned image representations, in which each data point corresponds to an ion image and is color‐coded by the manually annotated spatial pattern classifications used as ground truth (0–12). Representative ion images are also shown around the scatter plot and linked to their locations on the t‐SNE map. Adapted with permission.^[^ ^158^ ^]^ Copyright 2022, Royal Society of Chemistry.

Despite the success of these studies, they highlighted a challenge that the limited size of MSI data presents for conventional deep neural network training frameworks, which require a large amount of annotated ion images. Self‐supervised learning, which does not require any annotated data, is a promising approach for addressing this challenge. Hu et al.^[^ ^158^ ^]^ have developed a self‐supervised clustering approach for MSI spatial data compression using contrastive learning and image augmentation. Based on a simple framework for contrastive learning of visual representations (SimCLR) shown in **Figure** **7**A, a deep CNN encoder was trained using mouse uterine MSI data from a single experiment without manual annotations. This model was able to effectively learn high‐level spatial features from ion images and classify them based on molecular colocalizations, which are visualized in a 2D space using t‐SNE and color‐coded with ground‐truth spatial pattern classifications as shown in Figure 7B. The separation and compactness of clusters indicates the effectiveness of the CNN model at learning molecular localizations. Representative ion images shown around the t‐SNE plot further highlight the robustness of the model, which correctly classified ion images regardless of the signal and noise levels. In comparison with manual image annotation, this approach achieved >90% classification accuracy for the benchmark dataset and was successfully used to classify a MALDI imaging data set from METASPACE without any annotations. The self‐supervised learning approach may be incorporated into a semisupervised learning framework for training a larger deep learning model, which can generalize ion image classification for MSI data across tissue contexts and MSI instruments.

### 2.3 Data Interpretation

The ultimate goal of MSI data analysis is to obtain insights, which facilitate understanding of biochemical pathways. Statistical analysis is a key tool in MSI data interpretation. Combining MSI with other experimental modalities provides a unique opportunity to address specific scientific questions in the biological context. In addition, linking MSI data with knowledge bases, such as ontology and pathway databases enables the functional analysis of imaged molecules in the context of known biological processes. In this section, we review computational methods that have been used for MSI data interpretation in recent studies.

#### 2.3.1 Statistical Analysis

Statistical analysis methods are commonly used for comparison between different conditions examined in MSI experiments. Statistical tests assess the likelihood that MS peak intensities are different between sample cohorts. Although *t*‐test and analysis of variance (ANOVA) are the most widely used statistical tests,^[^ ^159^, ^160^, ^161^, ^162^, ^163^, ^164^ ^]^ other nonparametric tests that do not assume that data fits a Gaussian distribution including Mann‐Whiney U,^[^ ^165^, ^166^, ^167^, ^168^ ^]^ Wilcoxon rank‐sum,^[^ ^169^, ^170^ ^]^ Kolmogorov–Smirnov,^[^ ^171^ ^]^ and Kruskal–Wallis^[^ ^172^ ^]^ have also been used for MSI data interpretation. Based on the statistical test results, volcano plots are used to visualize ions with a statistically significant intensity difference between two groups, in which *p*‐value and fold‐change value are calculated for each ion.^[^ ^162^, ^163^, ^164^ ^]^

Statistical multivariate analysis is often used to discover biomarkers in MSI data. Discriminant analysis such as PLS‐DA and generalized linear model such as Lasso are most popular statistical methods. PLS‐DA uses a classical PLS regression where the response variable (Y) is categorical with respect to sample class membership. Based on class information, PLS‐DA linearly projects predictor variables (X) to maximize the discrimination between samples in different groups.^[^ ^167^, ^173^, ^174^, ^175^, ^176^ ^]^ The diagnostic statistics, Q^2^, is used to evaluate the statistical significance of PLS‐DA models. Variable influence on projection (VIP) usually guides the selection of predictor variables (ions).^[^ ^177^ ^]^ Meanwhile, Lasso is a multiclass logistic regression with L1 penalty. By shrinking the size of predictor variables, it selects a subset of MS signals in a model. Lasso weights in the model are also used to tentatively identify biologically relevant molecules.^[^ ^109^, ^110^, ^111^, ^112^, ^113^ ^]^ By taking into account a wide range of molecules, these powerful techniques may be used to identify potential biomarkers and establish statistical relationships between them.

The receiver operating characteristic (ROC) is usually performed for biomarker evaluation.^[^ ^25^, ^26^ ^]^ In ROC analysis of a univariate biomarker, samples are labeled into “positive” and “negative” classes. To evaluate one biomarker candidate, a discrimination threshold is varied in the range of its MS signals to separate the samples into two groups. As a result, a series of “true positive rate” (“sensitivity”) and “true negative rate” (“specificity”) are calculated with respect to threshold values. An ROC curve plots the “sensitivity” versus “1‐specificity”, and the area under the curve (AUC) indicates the predictive score. A high AUC value (0.8–1) in the ROC analysis is used for biomarker identification.^[^ ^25^, ^26^ ^]^ The ROC strategy has also been extended to multivariate biomarker analysis.

**Figure 8:**
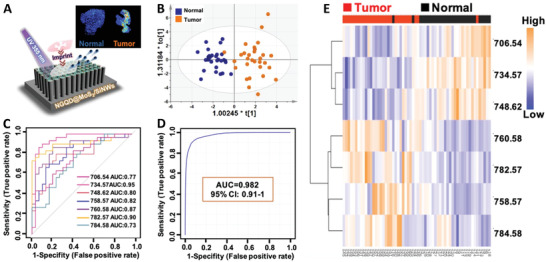
Statistical analysis of the MSI data of NSCLC tissues. A) An overview of the tissue imprinting MSI experiment. B) Orthogonal PLS‐DA score plots of metabolites from tumorous and normal tissues. ROC analysis for C) individual biomarkers and D) a panel of combined biomarkers. E) Heatmap and HC analysis of tumorous and normal tissues. Adapted with permission.^[^ ^176^ ^]^ Copyright 2022, American Chemical Society.

A combination of statistical tools is often used in MSI studies. For example, Liu et al.^[^ ^176^ ^]^ have developed a novel tissue imprinting method to image and diagnose nonsmall cell lung cancer (NSCLC) as shown in **Figure** **8**A. Using MSI data for both normal and cancer tissues, they developed an orthogonal PLS‐DA model to separate diseased and healthy tissues (Figure 8B). Based on the VIP values of molecules in PLS‐DA model and *p*‐values in *t*‐test, they selected a range of potential biomarkers of NSCLC. The ROC analysis confirmed that the univariate AUC values for these biomarker candidates are in the range of 0.73–0.95 (Figure 8C). Meanwhile, a panel of biomarkers shows an excellent discrimination between NSCLC and normal tissues with an AUC value of 0.982 (Figure 8D). The heatmap and HC analysis shown in Figure 8E further confirm that the identified biomarkers provide a clear differentiation between tumorous and normal tissues.

#### 2.3.2 Multimodal Integration

MSI provides an unprecedented depth of molecular information at the expense of the spatial resolution as compared to optical microscopy techniques. Data from other modalities provide complementary molecular or spatial information, which may be used to enhance the quality of MSI data or provide additional insights using more targeted analytical tools. MSI data have been coupled with histology,^[^ ^91^, ^133^, ^178^, ^179^, ^180^, ^181^, ^182^, ^183^, ^184^, ^185^, ^186^, ^187^ ^]^ fluorescence microscopy,^[^ ^188^, ^189^, ^190^, ^191^ ^]^ Allen brain atlas,^[^ ^107^, ^192^ ^]^ topology,^[^ ^193^, ^194^, ^195^ ^]^ electron microscopy,^[^ ^196^, ^197^ ^]^ Raman spectroscopy,^[^ ^198^, ^199^, ^200^, ^201^ ^]^ infrared spectroscopy,^[^ ^202^ ^]^ magnetic resonance imaging,^[^ ^93^, ^203^ ^]^ and microsampling LC‐MS/MS analysis of proteins, transcripts, and genes.^[^ ^164^, ^204^, ^205^, ^206^, ^207^, ^208^, ^209^, ^210^ ^]^ In these studies, computational approaches have been used for registration of the multimodal data.

In multimodal imaging experiments, analytical information for the same sample is recorded in different coordinate systems. Image registration finds a transformation that spatially aligns the information from one modality to another. Because of the differences in the spatial resolution and information depth, computational methods play a critical role in this analysis. Computational registration methods typically employ (a) a selection of feature space, (b) similarity measure, (c) transformation functions, and (d) parameter search strategy.^[^ ^211^ ^]^ For MSI data, fiducial markers or the whole image are usually selected as registration features. In MALDI experiments, laser ablation marks on tissue may be used as ground‐truth features for finding registration transformations.^[^ ^179^ ^]^ Pen marks drawn near the sample have also been used as markers for image registration.^[^ ^191^ ^]^ In addition, distinct geometrical features on tissues can be regarded as intrinsic markers. For example, the profiles of hippocampus, central canal, and cerebral peduncle in brain samples have been used to aid image registration of MSI data to the Allen brain atlas and histology images.^[^ ^107^, ^185^ ^]^ In the absence of fiducial markers, the geometrical information from the whole image may be used to facilitate registration. In conventional metrics for evaluating image similarity, such as mutual information, input images are required to have the same dimensionality.^[^ ^211^ ^]^ As a result, dimensionality reduction is typically applied to MSI data. For example, for registration with microscopy images that are usually saved in either the RGB or grayscale format, MSI data consisting of hundreds of images are compressed into an image with three or one channel, respectively. PCA,^[^ ^203^ ^]^ NMF,^[^ ^200^ ^]^ t‐SNE,^[^ ^91^, ^93^ ^]^ and UMAP^[^ ^184^, ^187^ ^]^ have been used to represent MSI data in a low‐dimensional space while retaining the original molecular information. The compressed MSI data are used to identify distinguishable anatomical features of tissues, which are subsequently employed to search for the optimal transformation function for image registration. After the selection of the registration feature spaces, one modality is used as a moving image and another as a fixed image. The coordinate system of the moving image is transformed to align its geometric features to those of the fixed image. In this computation, a registration algorithm iteratively searches transformation parameters by optimizing the similarity measure between two sets of features. Conventional linear and nonlinear transformations have been successfully applied to MSI data, including affine and b‐spline. Recently, a U‐Net CNN model has been used as the transformation function for registering MSI data to histology images, which provided a more robust registration than b‐spline for ion images containing background noise.^[^ ^183^ ^]^

**Figure 9:**
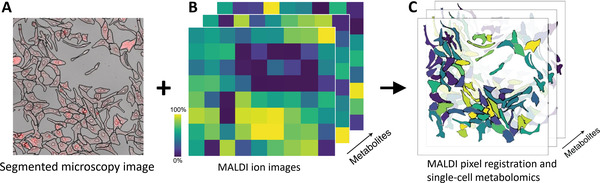
Multimodal integration workflow of SpaceM. A) Cell segmentation in light microscopy image. B) MALDI MSI for metabolite quantification. C) MALDI pixel registration and metabolite intensity normalization for single cells. Adapted with permission.^[^ ^191^ ^]^ Copyright 2021, Nature Publishing Group.

Ground‐truth labels for MSI pixels may be obtained by aligning well‐defined ROIs from other modalities to MSI data. Recently, this strategy has been used to link single cell identities to MS profiles.^[^ ^182^, ^191^ ^]^ For example, Rappez et al.^[^ ^191^ ^]^ have developed a SpaceM method to study single‐cell metabolomics by coupling MALDI imaging and light microscopy. By computationally processing the spatial overlap between MALDI ablation marks and cell segments (**Figure** **9**A), they assigned MS metabolite profiling data (Figure 9B) to each single cell, which enabled MS‐based metabolic characterization at a single‐cell level as shown in Figure 9C. Using this approach, the authors characterized metabolic heterogeneity of hepatocytes within a stimulated population. Another important computational method is image fusion, which refers to the generation of a single image from multimodal imaging data while providing a detailed description of the analyzed samples.^[^ ^211^ ^]^ Since the initial introduction of a predictive fusion method by Van de Plas et al.,^[^ ^178^ ^]^ image fusion has been used to enhance the spatial resolution of MSI data by generating imaging data from other modalities. In this approach, image fusion is performed as a multivariate PLS regression task, in which MS peak intensities are response variables and microscopy‐derived signals are predictor variables. By incorporating optical microscopy data into the constructed model, ion images with higher spatial resolution were predicted. Other computational methods that have been developed to sharpen MSI images based on complementary information from other modalities include pan‐sharpening algorithm,^[^ ^196^ ^]^ Laplacian pyramid fusion,^[^ ^197^ ^]^ patch‐based super resolution,^[^ ^180^ ^]^ and correspondence‐aware manifold learning.^[^ ^184^ ^]^ Other microsampling‐based multimodal approaches that do not require special computations are not highlighted here. For additional information on both method development and application of multimodal experiments, the readers are referred to detailed reviews.^[^ ^8^, ^19^, ^20^ ^]^

Different MSI modalities have been combined to obtain complementary molecular and spatial information for the same sample.^[^ ^212^, ^213^, ^214^, ^215^, ^216^, ^217^, ^218^, ^219^, ^220^, ^221^, ^222^, ^223^, ^224^ ^]^ For example, SIMS and MALDI have been used to obtain correlative imaging data of cholesterol and other small molecules with proteins.^[^ ^212^ ^]^ Meanwhile, DESI and MALDI have been used in sequence on the same tissue section to obtain spatial maps of lipids and proteins.^[^ ^213^ ^]^ Correlative imaging using soft ionization techniques that provide molecular information with elemental mapping using laser ablation‐inductively coupled plasma (LA‐ICP) has been used for studying the effect of platinum‐based therapy in colorectal and ovarian cancer.^[^ ^214^ ^]^ Several computational tools have been developed to enable integration and analysis of multimodal MSI data. For example, Borodinov et al.^[^ ^219^ ^]^ developed a strategy to sputter fiducial markers using focused ion beam to enable data integration of SIMS and MALDI data. Computational coregistration was achieved by marker searching and transformation matrix optimization. They further used NMF and canonical correlation analysis (CCA) to reconstruct high spatial resolution MALDI molecular images by correlating them with SIMS data. Meanwhile, Castellanos‐Garcia et al.^[^ ^223^ ^]^ used t‐SNE MSI data compression to automatically register LA‐ICP images to MALDI images acquired without fiducial markers. This approach may be used to correlate molecular information provided by MALDI MSI with elemental information provided by LA‐ICP‐MSI. After the spatial alignment, multiple algorithms, including random forest,^[^ ^215^, ^217^ ^]^ PCA, and CCA,^[^ ^212^ ^]^ have been applied to obtain complementary imaging information from different modalities.

#### 2.3.3 Reference Knowledge Base

**Figure 10:**
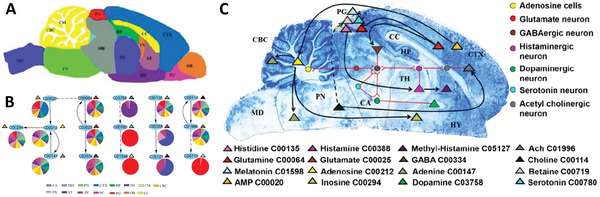
A) Mouse brain anatomical segmentation map. B) Metabolic pathway and related distributions of neurotransmitters and their related metabolites in rat brain visualized in linked pie charts. C) The neurotransmitter regulated and metabolic networks in mouse brain. Adapted with permission.^[^ ^228^ ^]^ Copyright 2021, American Chemical Society.

In the early days of MSI data analysis, database search approaches were only used for annotating MS peaks. More recently, MSI data have been combined with reference knowledge base to obtain a better understanding of the function of imaged molecules in biological systems. Gene and lipid ontology enrichment analyses through database searching have been used to examine the metabolic states of mouse blastocyst implantation^[^ ^225^ ^]^ and the stimulation of human hepatocytes with fatty acids,^[^ ^191^ ^]^ respectively. Molecular pathway analyses have been used to derive biological insights from MSI data. For example, the Kyoto encyclopedia of genes and genomes (KEGG) database^[^ ^226^, ^227^, ^228^ ^]^ and computational search tools, such as Reactome^[^ ^229^ ^]^ and MetaboAnalyst,^[^ ^230^ ^]^ have been used to construct metabolic pathways based on MSI data. Protein pathways and lipid–protein interactions have also been examined using Reactome,^[^ ^120^ ^]^ STING,^[^ ^231^ ^]^ and MetaCore^[^ ^232^ ^]^ analysis of MSI data. Molecular pathways with rich functional information are usually organized into networks for the efficient visualization of biochemical pathways that contribute to the observed spatial localization of molecules in tissue samples. Such integration of MSI data with reference knowledge base provides important insights into key biochemical pathways in a system and their response to alterations. As discussed earlier, networking analysis has also been used to represent MSI spectral or spatial information.^[^ ^77^, ^145^ ^]^ Several studies focused on the development of computational methods that use spatially resolved molecular information provided by MSI to understand the functional state of a biological system by constructing a spatially resolved network of metabolic pathways. For example, Pang et al.^[^ ^228^ ^]^ built a metabolic network for a rat brain tissue using MetaboAnalyst based on KEGG database with peak information from an air flow‐assisted DESI MSI experiment. Using image segmentation shown in **Figure** **10**A, they combined neurotransmitters and related metabolites (nodes) into a network with individual pie charts showing their distributions in different brain regions (Figure 10B). This network was then spatially mapped onto a microscopy image, which visualizes neuron regulation and metabolic reactions in a rat brain as shown in Figure 10C. This approach provided first insights into metabolic dysregulation in a scopolamine‐treated Alzheimer's rat model.

### 2.4 MSI Data Analysis Software

As described earlier, computational methods are essential tools for data mining and visualization of MSI data. Several commercial and open‐source software including SCiLS, Peak‐by‐Peak,^[^ ^18^ ^]^ MSI QuickView,^[^ ^233^ ^]^ MSiReader,^[^ ^234^ ^]^ OpenMSI,^[^ ^235^ ^]^ OmniSpect,^[^ ^236^ ^]^ msIQuant,^[^ ^237^ ^]^ MassImager,^[^ ^238^ ^]^ METASPACE,^[^ ^32^ ^]^ LipostarMSI,^[^ ^239^ ^]^ and MassExplorer^[^ ^240^ ^]^ have implemented these computational algorithms and workflows in graphical user interfaces. Functions and features of these software programs are summarized in **Table** **1**. Most programs support open‐source data formats such as imzML and mzXML with only some software providing direct access to raw data files saved in vendor‐specific formats. Several standalone converters, such as ProteoWizard msconvert, may be used for data conversion into the imzML format compatible with a majority of MSI analysis software programs. After data importing and preprocessing, MSI data are usually organized into a universal data cube structure as shown in Figure 4 for further visualization and multivariate analysis. Among these software packages, METASPACE is a unique cloud platform, which enables users to perform data annotation, visualization, and management via a browser. In addition, it is the largest community‐populated knowledge base of MSI data. With valuable ground‐truth data provided by experts, AI approaches have been developed and integrated into the platform. In another trend, an increasing number of MSI researchers adopt programming tools such as R and Python for data analysis. Because of the complexity of MSI experiments, multiple computational methods are usually combined to construct data analysis pipelines, which are not always available in the existing graphical software programs. Open‐source packages and modules provide an opportunity to implement and test novel computational tools. Many open‐source packages for MSI data analysis have been developed including MALDIquant,^[^ ^241^ ^]^ Cardinal,^[^ ^242^ ^]^ rMSI,^[^ ^243^ ^]^ massPix,^[^ ^244^ ^]^ BASTet,^[^ ^245^ ^]^ SPUTNIK,^[^ ^246^ ^]^ rMSIproc,^[^ ^247^ ^]^ IM‐MSIC,^[^ ^248^ ^]^ and HIT‐MAP.^[^ ^69^ ^]^ The functions and features of these packages are summarized in **Table** **2**. Github offers an impressive repository of novel computational tools in an open‐source format, which facilitates the implementation and communication of new ideas. In addition, Python and R are currently mainstream programming languages for data science, statistics, and computer vision. Building workflows in these ecosystems makes it possible to swiftly introduce approaches from other fields into MSI data analysis.

**Table 1:** Popular software for analyzing MSI data

Software	Environment	License	Vendor raw files	Preprocessing	Visualization	Multivariate analysis	Molecular annotation
SCiLS	Standalone application	Proprietary	✓	✓	✓	✓	
Peak‐by‐Peak	Standalone application	Proprietary	✓	✓	✓		
MSI QuickView	Standalone application	Open source		✓	✓		
MSiReader	Standalone application	Open source		✓	✓	✓	
OpenMSI	Standalone/web‐based application	Proprietary		✓	✓	✓	
OmniSpect	Standalone application/web‐based application	Open source		✓	✓	✓	
msIQuant	Standalone	Proprietary		✓	✓		
MassImager	Standalone application	Proprietary		✓	✓	✓	
METASPACE	Web‐based application	Open source		✓	✓		✓
LipostarMSI	Standalone application	Proprietary	✓	✓	✓	✓	✓
MassExplorer	Standalone/web‐based application	Open source		✓	✓	✓	

**Table 2:** Popular software packages for analyzing MSI data

Package	Programming language	Open source	Vendor raw files	Preprocessing	Visualization	Molecular annotation	Customized multivariate analysis	Note
MALDIquant	R	✓		✓	✓			
Cardinal	R	✓		✓	✓		✓	
rMSI	R	✓		✓	✓			
massPix	R	✓		✓	✓	✓	✓	Specialized in lipidomics
BASTet	Python	✓		✓	✓		✓	
SPUTNIK	R	✓		✓	✓		✓	Specialized in peak filtering
rMSIproc	R	✓		✓	✓			
IM‐MSIC	Python	✓		✓	✓			Specialized in ion mobility MSI
HIT‐MAP	R	✓		✓	✓	✓	✓	Specialized in MALDI‐MSI Proteomics

## 3 Computational Methods for MSI Experiments

**Figure 11:**
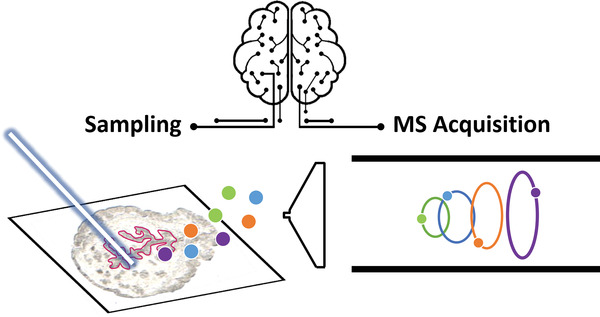
Computational methods enable “smart” sampling and MS acquisition.

Since the initial introduction of the MSI technology, the development of analytical capabilities in MSI has been driven by advancements in the ionization sources and mass spectrometry instrumentation. In recent years, computational methods have emerged as an enabling technology to further enhance the throughput and molecular coverage of MSI experiments. Without requiring major modifications to the hardware, computational approaches are used to guide sampling and acquisition modules of MSI platforms as illustrated in **Figure** **11**.

### 3.1 “Smart” Sampling

In a conventional MSI experiment, a manually selected tissue region is uniformly sampled and data acquisition by a mass spectrometer is performed at a constant rate. As a result, an increase in the spatial resolution usually results in the quadratic growth of data acquisition time. For example, state‐of‐the‐art commercial MALDI instruments operated at the acquisition rate of 40 pixels s^−1^ enables imaging of a 1 cm^2^ sample in 17 min with a step size of 50 µm and 7 h with a step size of 10 µm. Several “smart” sampling computational methods have been developed to address this throughput limitation of MSI experiments.

**Figure 12:**
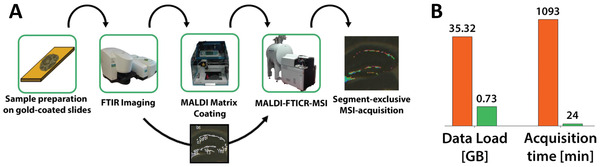
FTIR‐guided, spatially restricted data acquisition by high‐resolution MALDI‐FTICR‐MSI. A) A multimodal workflow for the consecutive FTIR and MALDI FT‐ICR MSI experiments on a single slide. B) Selective MSI acquisition of the granular cell layer found in the hippocampus of a sagittal mouse brain section significantly decreases data load (97.9%) and acquisition time (97.8%). Red and green bars represent whole tissue and selective MSI imaging, respectively. Adapted under the terms of the Creative Commons Attribution 4.0 International License.^[^ ^253^ ^]^ Copyright 2018, The Authors, published by Springer Nature.

One type of smart sampling methods relies on combining different imaging modalities. In this approach, tissues are analyzed using a fast imaging modality prior to MSI experiments. Then, the geometrical information from the first imaging experiment is computationally processed to spatially guide MSI sampling. Selective sampling of key regions of the sample identified using one modality reduces data acquisition time of MSI experiments while providing the same spatial resolution. Optical microscopy,^[^ ^249^ ^]^ histology,^[^ ^250^, ^251^ ^]^ polarimetry,^[^ ^252^ ^]^ and infrared spectroscopy^[^ ^253^ ^]^ have been used to localize tumors or other spatial patterns on tissue for subsequent MSI analysis. Selected ROIs are computationally segmented and registered to the second experimental coordinates for guiding MSI acquisition. For example, Rabe et al.^[^ ^253^ ^]^ developed a workflow to exploit nondestructive Fourier transform infrared (FTIR) microscopy to automatically guide high‐resolution MALDI MSI data acquisition as shown in **Figure** **12**A. Based on the FTIR imaging data, they segmented anatomical structures of mouse brain tissues using a clustering algorithm and aligned them to the MSI coordinate system. As a result, dentate gyrus of the hippocampus was localized and selectively imaged by MSI experiments, which reduced the acquisition time and data size by more than 97% (Figure 12B) as compared to imaging of the entire sample. This strategy has been recently used for the high‐throughput single‐organelle characterization with subcellular precision.^[^ ^254^ ^]^

**Figure 13:**
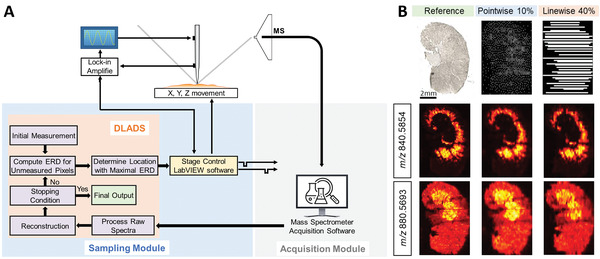
High‐throughput MSI with dynamic sparse sampling. A) An overview of the dynamic sparse sampling nano‐DESI MSI platform. B) DLADS sparse sampling and reconstruction of ion images. An optical image of mouse kidney tissue and ground‐truth ion images of *m*/*z* 840.5854 and *m*/*z* 880.5693 are shown in the first column. DLADS sampling locations and reconstructed ion images for 10% pointwise and 40% linewise measurements are shown in the columns 2–3. Adapted under the terms of the CC‐BY License.^[^ ^257^ ^]^ Copyright 2022, The Authors, published by American Chemical Society.

Compressed sensing is another “smart” sampling strategy that does not require external input. Compressed sensing is an approach, which relies on sparse sampling from selected locations on a sample followed by image reconstruction using a uniform virtual grid. Xie et al.^[^ ^255^ ^]^ have integrated a compressed sensing approach with subspace modeling to accelerate FT‐ICR MALDI MSI experiments. In this work, a joint subspace and spatial sparsity constrained model enabled computational reconstruction of high‐resolution MSI images using short FT‐ICR transient signals from randomly sampled pixels covering 40% of the sample. In another development, sampling is guided to molecularly informative locations on a sample, which further enhances the MSI imaging throughput and improves the fidelity of image reconstruction. For example, Helminiak et al.^[^ ^256^ ^]^ have developed a deep learning approach for dynamic sampling (DLADS), which dynamically determines molecularly informative sampling locations using a CNN model. A combined experimental and computational study by Hu et al. evaluated DLADS in both pointwise and linewise acquisition modes representing MALDI and nano‐DESI experiments, respectively.^[^ ^257^ ^]^ The experimental platform, in which DLADS is coupled with nano‐DESI MSI through hardware and software integration is illustrated in **Figure** **13**A. In each iteration, DLADS determines molecularly informative locations and interfaces with both the XYZ stage control and data acquisition software to direct data acquisition to these locations. The sampled locations provided by DLADS are shown in the top row of Figure 13B and ion images for two representative *m*/*z* reconstructed from sparse MSI data are shown in the bottom row of Figure 13B. An excellent correspondence is observed between reconstructed and ground truth ion images shown in the first column. This approach has been used to obtain a 2.3‐fold improvement in the experimental throughput of nano‐DESI MSI and is estimated to provide a tenfold improvement for pointwise acquisition based on simulation results.

### 3.2 “Smart” MS Data Acquisition

Computational methods have been also used for “smart” data acquisition, which improves the throughput, molecular specificity, and coverage of MSI experiments. For example, Xie et al.^[^ ^258^ ^]^ have developed a subspace approach to accelerate the experimental throughput of FT‐ICR MSI. They demonstrated that the measured FT‐ICR transients may be projected onto a lower‐dimensional subspace with minimal loss of fidelity. Thus, they reduced the experimental time for FT‐CIR MSI imaging by acquiring both long transients for 10% of all the pixels and truncated transients for 90% all the pixels in the same experiment. High‐fidelity ion images are reconstructed by estimating basis transients and spatial coefficients from long transients and reconstructing long transients from short transients acquired experimentally using estimated parameters. Ellis et al.^[^ ^259^ ^]^ achieved MS/MS based molecular identification in each pixel using an automated acquisition approach. Using MALDI coupled to an ion trap‐Orbitrap instrument, they synchronized the MALDI stage with the parallel Orbitrap intact ion acquisition and ion trap MS/MS acquisition. This strategy enabled data‐dependent acquisition of MS/MS data for precursor ions observed in the Orbitrap in every adjacent pixel location without sacrificing imaging throughput. They also built a data pipeline to interpret molecular images using both Orbitrap intact ion distribution and MS/MS ion annotations. Su et al.^[^ ^260^ ^]^ developed a proteoform imaging mass spectrometry method by coupling nano‐DESI MSI with individual ion mass spectrometry (I^2^MS).^[^ ^261^ ^]^ Using Orbitrap‐based charge detection I^2^MS and computational signal transformation, 169 proteoforms have been identified in every pixel and proteoform‐selective imaging data were obtained with a spatial resolution of 80 µm.

## 4 Conclusion and Outlook

The unprecedented chemical specificity and untargeted, label‐free molecular imaging capabilities of MSI uniquely position it as a powerful technique for biological research, drug discovery, clinical research, natural product discovery, and biomedical applications. Recent advances in the development of this technology present several challenges and opportunities for computational tools. In particular, computational methods are currently developed for enhancing MSI capabilities, delivering biomedical insights, and transforming data analysis workflows. For example, automated approaches for spectral processing, feature detection, and image segmentation have been incorporated into routine analysis workflow for large and complex MSI data. Statistical analysis has become an essential tool for evaluating the quality of MSI data and discovering biomarkers. In addition, multimodal analysis involving MSI has been used to precisely target important biological questions. We anticipate that the role of computational methods in MSI will continue to grow providing the infrastructure and enabling technologies for new applications and scientific discoveries.

Future development of advanced molecular annotation approaches will significantly improve the interpretability of MSI data. For example, coupling of MSI with ion mobility or MS/MS without sacrificing imaging throughput may be used to improve molecular coverage and facilitate identification. The development of efficient database searching and AI‐based model predictions for annotating molecules using multimodal imaging data will play an important role in streamlining data interpretation. Community resources, such as METASPACE, METLIN, and GNPS will facilitate molecular annotations and provide high‐quality labeled data for training of predictive models. Confident annotations will facilitate pathway and ontology analyses. Combining this information with knowledge graphs in a spatially resolved manner presents a unique opportunity for obtaining biological insights from MSI data.

The revolution in other fields, such as AI, computer vision and cloud computing, will continue to inspire computational research in MSI. Self‐supervised learning, which enables training of a sophisticated model without requiring large, labeled data will provide better ion image reconstruction along with spectral and image classification than traditional approaches. The development of public MSI repositories containing high‐quality curated MSI data will provide benchmark datasets similar to ImageNet in deep learning for evaluating computational methods and developing generic models across biological contexts and instrument types. Cloud resources have democratized computational studies for researchers who cannot afford investing in expensive computing hardware. In the future, cloud platforms will play a more important role in MSI data management, visualization, processing, and reporting. It is expected to become a readily accessible tool for every MSI researcher equipped with a browser application installed on a personal computer.

The trend of open science will continue to accelerate MSI research. Currently, most mass spectrometer vendors provide open‐source libraries for transparent access to raw MS data, which has enabled the development of open‐source MSI data analysis software packages and pipelines. The next revolution will be in the development of the open‐source hardware for MSI experiments including solutions for motorized computer‐controlled sample stages, ionization sources, and data acquisition strategies. For example, several MS vendors provide access to instruments APIs, which has been used by the community to develop solutions for the automated optimization of MS acquisition parameters.^[^ ^262^ ^]^ Open‐source hardware will further enable researchers to implement data‐driven experiments and expand MSI capabilities. Computational methods are expected to play an important role in these developments.

## Conflict of Interest

The authors declare no conflict of interest.
